# Genomic Modeling of an Outbreak of Multidrug-Resistant *Shigella sonnei*, California, USA, 2023–2024

**DOI:** 10.3201/eid3113.241307

**Published:** 2025-05

**Authors:** Tyler Lloyd, Sana M. Khan, Dustin Heaton, Munira Shemsu, Vici Varghese, Jay Graham, Misha Gregory, Penny Dorfman, Megan Talton, Jessica DeVol, Nicola F. Müller, Kavita K. Trivedi

**Affiliations:** Alameda County Public Health Department, San Leandro, California, USA (T. Lloyd, S.M. Khan, D. Heaton, M. Shemsu, V. Varghese, M. Gregory, P. Dorfman, M. Talton, J. DeVol, K.K. Trivedi); University of California, Berkeley, California, USA (T. Lloyd, J. Graham); Centers for Disease Control and Prevention, Atlanta, Georgia, USA (S.M. Khan); University of California, San Francisco, California, USA (N.F. Müller)

**Keywords:** *Shigella sonnei*, bacteria, antimicrobial resistance, multidrug resistance, epidemiology, phylodynamic modelling, outbreak, California, United States

## Abstract

We report the detection of a *Shigella sonnei* outbreak from a small investigation in the San Francisco Bay area, California, USA, in 2024. By combining outbreak investigation with genomic sequencing, we show the utility of phylodynamics to aid outbreak investigations of bacterial pathogens by state or local public health departments.

In January 2024, a board and care facility (facility A) in the San Francisco Bay area, California, USA, reported 4 cases of *Shigella sonnei* infection to the Alameda County Public Health Department (ACPHD; San Leandro, California, USA). Shigellemia was confirmed in 3 patients. In February 2024, an independent living center (facility B) reported 3 cases of *Shigella* infection. *Shigella* bacteremia was confirmed in 2 patients (Figure 1,2). *Shigella* bacteremia (shigellemia) is rare but associated with immature immune responses or immunocompromised adults (*1*). We performed a 10-year retrospective review of *Shigella* cases in Alameda County and found 0.7% of cases had positive blood samples reported, consistent with other reviews on *Shigella* bacteremia (*2*). The 7 cases from 2 facilities prompted patient investigations at facilities A and B, and investigations into other *S. sonnei* patients in Alameda County during December 2023–February 2024.

**Figure 1:**
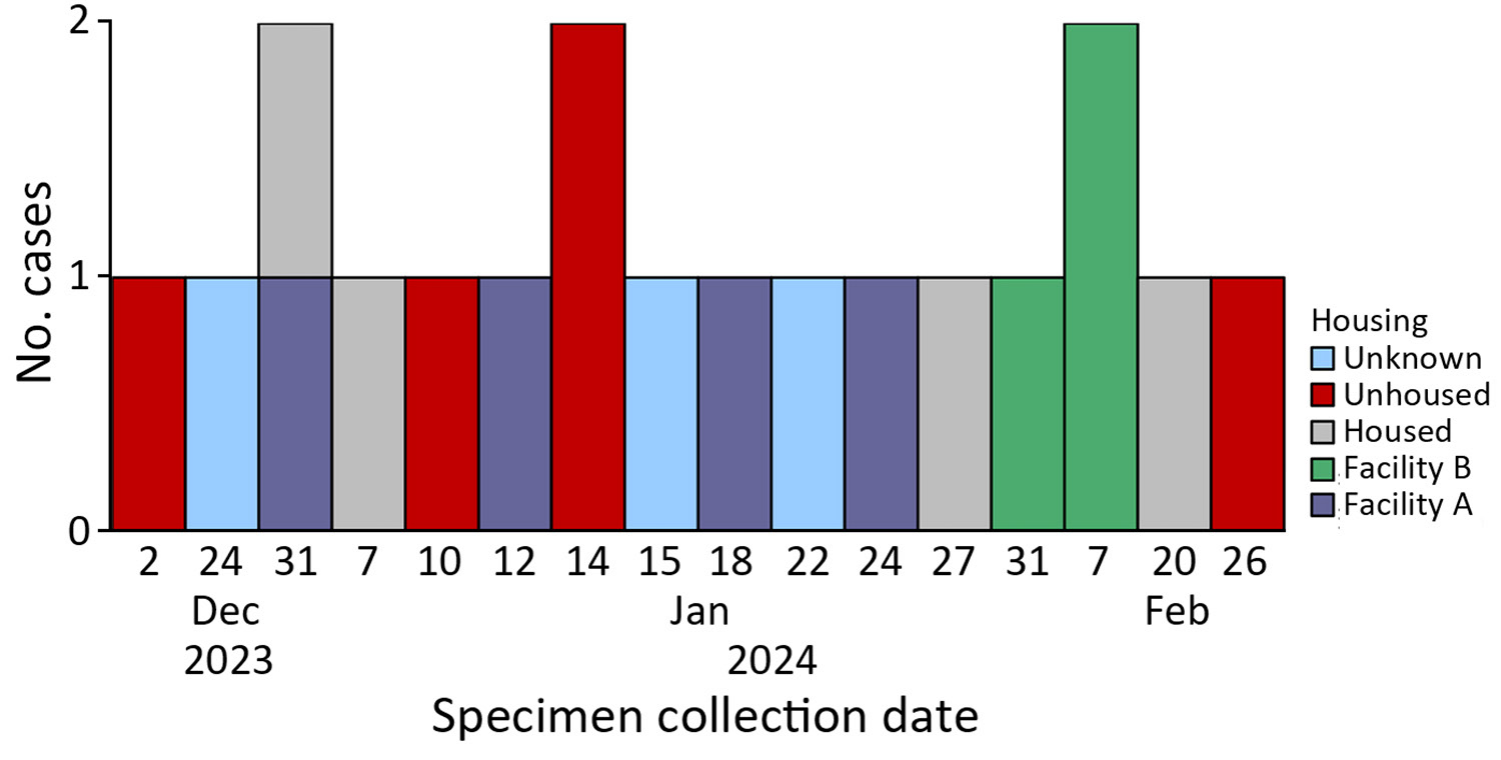
Epidemiologic curve showing specimen collection dates of shigellemia cases and housing status in *Shigella sonnei* case investigation with known linkages, California, USA, 2023–2024.

**Figure 2:**
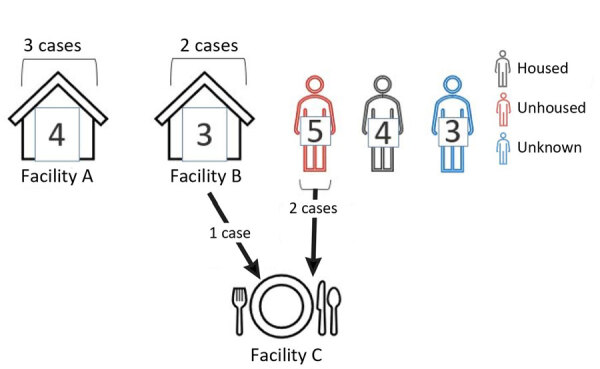
Diagram showing the number of cases from linked facilities, the housing status of patients, and the linkage between cases in *Shigella sonnei* case investigation with known linkages, California, USA, 2023–2024.

## Methods and Materials

Case investigations were limited but included symptom onset, severity, housing status, and other attainable risk factors. The outbreak investigation linked patients from facility B and 2 unhoused community members to a third location (facility C) where marginally housed community members gather. No clear transmission pattern was determined through epidemiologic investigation. We identified 19 genotypically identical *S*. *sonnei* isolates during December 2, 2023–February 26, 2024, among all cases in facilities A, B, and C.

Of the 19 patients, 13 (68%) were male and 6 (32%) female; median age was 59 years, and 9 (47%) were White and 10 (52%) non-Hispanic. Case investigations were completed on 16 of the 19 patients; 5 (26%) were experiencing homelessness, 4 (21%) were associated with facility A, 3 (15%) were associated with facility B, 4 (21%) had stable housing, and 3 (15%) had unknown housing. Drug use history was known in 3 patients. Of the 5 patients with shigellemia, 1 reported drug use. Sexual contact was unknown or denied during the incubation period for all patients. All treatment regimens where data were available were appropriate for the antimicrobial drug susceptibility data; all patients recovered.

Of the 3 patients associated with facility C, 2 were experiencing homelessness and 1 volunteered as a food handler at facility C while ill. The third patient from facility B visited facility C and had symptoms develop 14 days after exposure to the food handler at facility C. No other epidemiologic links were established among the 19 cases. No comorbidities were found in electronic medical records. However, determining precise risk factors in patients experiencing homelessness, such as where they sheltered during their infectious period, contact with each other, using the same resources, public restrooms or transportation, was not possible.

## Results

Whole-genome sequencing (WGS) of *Shigella* isolates revealed highly similar sequences, suggesting an epidemiologic link. The time from notification of a potential outbreak in facility A to WGS confirmation was 8 days. We genotyped the isolates, which belonged to genotype 3.7.26, as previously described (*3*). This method removes repetitive regions with higher rates of potentially erroneous single-nucleotide polymorphisms (SNPs). The method simplified interpretation by providing a numerical genotype, making it easy to determine close ancestry and it was part of our routine bioinformatics workflow (*4*). References for genotype 3.7.26 are from the United Kingdom (2013) and France (2014). We confirmed phenotypic multidrug resistance by using antimicrobial drug resistance gene detection (Appendix).

**Figure 3:**
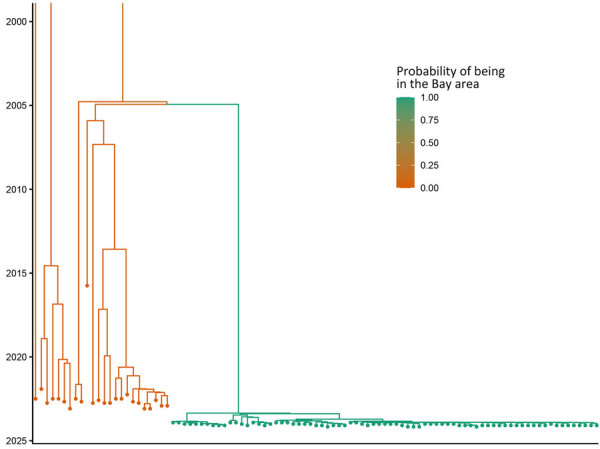
Maximum clade credibility tree of outbreak samples and contextualized *Shigella sonnei* isolates from the United States, 2015–2023. The color scale denotes the probability of the node below each branch being in the San Francisco Bay, California, USA, area. Inferred by using MASCOT (*6*).

**Figure 4:**
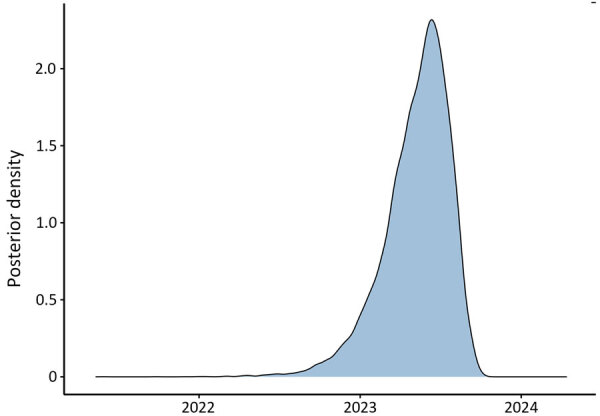
Distribution curve of the predicted dates of the most recent common ancestor of the *Shigella sonnei* outbreak isolates, California, USA, 2023–2024. The plot shows the posterior density for the common ancestor times of the *Shigella* sequences collected in the San Francisco Bay, California, USA, area. For a single introduction, the common ancestor time provides a lower bound on the timing of the introduction into the San Francisco Bay area.

**Figure 5:**
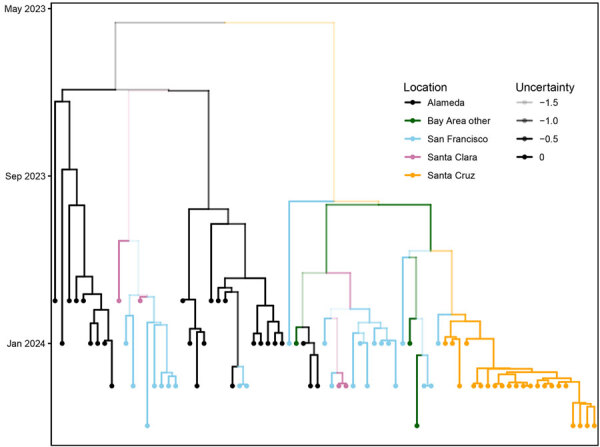
Phylogenetic tree of *Shigella sonnei* outbreak isolates in the San Francisco Bay, California, USA, area with spatiotemporal metadata and tree uncertainty, 2023–2024. Branches are colored according to location. The opacity of the branches is equal to the uncertainty of the placement of each branch. Phylodynamic methods are incorporated into phylogenetic trees with time and location.

During the retrospective sequencing of *S*. *sonnei* from patients treated in Alameda County, we identified patients with highly similar isolates in neighboring counties. The lack of specific links in the investigation and detection of cases from neighboring counties prompted ACPHD to notify the California Department of Public Health in March 2024, which led to a prioritization of *Shigella* isolates for sequencing. A total of 75 genetically related isolates were identified by California Department of Public Health by using PulseNet whole-genome multilocus sequence typing (MLST) (*5*), which showed relatedness but did not incorporate metadata. To reconstruct the spatial transmission dynamics of the outbreak, we performed a time-resolved, phylogeographic method known as the marginal approximation of the structured coalescent (MASCOT-skyline) in collaboration with the University of California, San Francisco. This approach uses Bayesian inference to reconstruct spatiotemporal transmission of pathogens and is implemented in the open-source program BEAST2 (*6*). MASCOT-skyline incorporates sampling time and sampling location of isolates. MASCOT-skyline then infers a posterior estimate of where the bacterial lineage was in the past. From this result, we inferred that all isolates were the result of a single introduction into the area (*7*; N.F. Müller, et al., unpub. data, https://pmc.ncbi.nlm.nih.gov/articles/PMC10942421). We obtained the molecular clock rate by contextualizing outbreak samples with 24 *S. sonnei* Pulsenet sequences from 2015–2023 to ensure an appropriate number of samples and the time span to effectively estimate the clock rate. The mean clock rate for the core-genome SNP alignment (length 1491 bp) was 3.341 × 10^−3^ substitutions per site per year. The sequences formed a distinct cluster from all other historical sequences (Figure 3). We inferred time to most recent common ancestor was most likely June 2023 (95% CI November 2022–August 2023), providing an upper bound on the time of introduction (Figure 4). We analyzed the outbreak at a more granular scale by using the same regional alignment and fixing clock rate while removing contextual sequences. We found the samples were geographically clustered by county within the outbreak (Figure 5).

One patient was a food handler at facility C, but no evidence of foodborne transmission was found. The shigellemia cases prompted us to investigate this cluster; however, patients were immunocompetent, and virulence markers were identical to nonbloodstream infections. Host factors such as intravenous drug use and sexual contact were incomplete and remain possible factors for shigellemia. 

## Discussion

The advent of phylodynamic approaches and genomic epidemiology has provided public health with additional insight into the spread of diseases, transmission chains, and mutations when using laboratory data paired with epidemiologic information. In this article, we demonstrate the use of phylodynamic modeling alongside a traditional case investigation to better determine outbreak dynamics and inform public health actions. Bacterial genomic epidemiology has historically relied on MLST, SNPs, whole-genome MLST, or a combination of technologic tools. However, those tools do not enable us to characterize the direction and timing of disease spread. SNP cutoff levels have shown variable specificity and sensitivity in identifying closely related bacterial isolates (*8*).

We also describe the role of the local public health laboratory to initiate enhanced WGS of *S. sonnei* to discover unlinked cases and identify a regional outbreak. We describe the timeline of the outbreak identification, notification of the state public health department, and phylodynamic methods to provide evidence of a single introduction and incorporate metadata into bacterial genomic epidemiology. However, those models do not guarantee complete ascertainment of transmission, and the inability to gather complete data on risk factors to link specific case manifestations, symptoms, or other factors associated with shigellemia or its mode of transmission is a limitation of our study. Ideally, genomic sequencing paired with epidemiologic information gathered, such as case manifestation, risk factors identified, and symptoms, can provide improved insights into the drivers of transmission. This information can be particularly helpful when investigating outbreaks in communities such as persons experiencing homelessness, when epidemiologic information may be limited. We recommend public health prevention measures focus on the proper maintenance, routine disinfection, and cleaning of public restroom facilities and handwashing stations, particularly in places that are frequented by persons experiencing homelessness.

Appendix Additional information about genomic modeling of an outbreak of multidrug resistant *Shigella sonnei*, California, USA, 2023–2024.
