# Supplementary material for: Genomic Modeling of an Outbreak of Multidrug-Resistant Shigella sonnei, California, USA, 2023–2024
Source: Emerg Infect Dis. 2025 May;31(Suppl 1):S98–S102. doi: 10.3201/eid3113.241307 (PMC12078535; doi:10.3201/eid3113.241307)
Supplement: Appendix — Additional information about genomic modeling of an outbreak of multidrug resistant Shigella sonnei, California, USA, 2023–2024. [file 24-1307-Techapp-s1.pdf]

Article DOI: https://doi.org/10.3201/eid3113.241307

EID cannot ensure accessibility for supplementary materials supplied by authors. Readers who have difficulty accessing supplementary content should contact the authors for assistance.

Genomic Modeling of an Outbreak of Multidrug Resistant *Shigella sonnei*, California, USA, 2023–2024

Appendix

Methods

We sequenced all isolates on an Illumina MiSeq using v2 300 cycle chemistry in a paired end, 2x150bp configuration. All raw sequence data fastq files were assembled using SPAdes v 1.1, and genotyping was performed using sonneityping in mykrobe v 0.12.1 (*1*,*2*). To determine the presence of antibiotic gene resistance, gene annotation was performed using ResFinder with database v 4.11.1 and AMRFinder Plus v 3.11.20 (*3–5*). Using these databases, we did not identify resistance genes unique to the cases of bacteremia. Genes for the specific antimicrobial classes that help to predict resistance were: cephalosporins (*blaCTX-M-15*), tetracyclines (*tet(A)*), aminoglycosides (*aph (6)-Id*, *aph(3")-Ib, aadA1),* trimethoprim (*drfA1*), and quinolones (*qnrS1*)." Variant calling was performed using snippy v 4.6.0 using reference genome NC_007384.1 (*5*). BEAST2 xml parameters, and R code for generating trees can be found in this github respository www.github.com/tjlloyd/ShigellaMASCOT.

References

1. Bankevich A, Nurk S, Antipov D, Gurevich AA, Dvorkin M, Kulikov AS, et al. SPAdes: a new genome assembly algorithm and its applications to single-cell sequencing. J Comput Biol. 2012;19:455–77. PubMed https://doi.org/10.1089/cmb.2012.0021

2. Hawkey J, Paranagama K, Baker KS, Bengtsson RJ, Weill FX, Thomson NR, et al. Global population structure and genotyping framework for genomic surveillance of the major dysentery pathogen, *Shigella sonnei.* Nat Commun. 2021;12:2684. PubMed https://doi.org/10.1038/s41467-021-22700-4

3. Bortolaia V, Kaas RS, Ruppe E, Roberts MC, Schwarz S, Cattoir V, et al. ResFinder 4.0 for predictions of phenotypes from genotypes. J Antimicrob Chemother. 2020;75:3491–500. PubMed https://doi.org/10.1093/jac/dkaa345

4. Camacho C, Coulouris G, Avagyan V, Ma N, Papadopoulos J, Bealer K, et al. BLAST+: architecture and applications. BMC Bioinformatics. 2009;10:421. PubMed https://doi.org/10.1186/1471-2105-10-421

5. Feldgarden M, Brover V, Gonzalez-Escalona N, Frye JG, Haendiges J, Haft DH, et al. AMRFinderPlus and the reference gene catalog facilitate examination of the genomic links among antimicrobial resistance, stress response, and virulence. Sci Rep. 2021;11:12728. PubMed https://doi.org/10.1038/s41598-021-91456-0

6. Seeman T. Snippy: rapid haploid variant calling and core genome alignment. Github Repository. [cited 2025 Feb 11]. https://github.com/tseemann/snippy
